# AP-1/KIF13A Blocking Peptides Impair Melanosome Maturation and Melanin Synthesis

**DOI:** 10.3390/ijms19020568

**Published:** 2018-02-14

**Authors:** Cécile Campagne, Léa Ripoll, Floriane Gilles-Marsens, Graça Raposo, Cédric Delevoye

**Affiliations:** 1 Institut Curie, PSL Research University, CNRS, UMR144, Structure and Membrane Compartments, F-75005 Paris, France; cecile.campagne@curie.fr (C.C.); Lea.ripoll@curie.fr (L.R.); floriane.gilles@univ-lyon1.fr (F.G.-M.); graca.raposo@curie.fr (G.R.); 2 Institut Curie, PSL Research University, CNRS, UMR144, Cell and Tissue Imaging Facility (PICT-IBiSA), F-75005 Paris, France

**Keywords:** pigmentation, melanocytes, kinesin, adaptor protein, peptide, melanosome biogenesis and maturation

## Abstract

Melanocytes are specialized cells that generate unique organelles called melanosomes in which melanin is synthesized and stored. Melanosome biogenesis and melanocyte pigmentation require the transport and delivery of melanin synthesizing enzymes, such as tyrosinase and related proteins (e.g., TYRP1), from endosomes to maturing melanosomes. Among the proteins controlling endosome-melanosome transport, AP-1 together with KIF13A coordinates the endosomal sorting and trafficking of TYRP1 to melanosomes. We identify here β1-adaptin AP-1 subunit-derived peptides of 5 amino acids that block the interaction of KIF13A with AP-1 in cells. Incubating these peptides with human MNT-1 cells or 3D-reconstructed pigmented epidermis decreases pigmentation by impacting the maturation of melanosomes in fully pigmented organelles. This study highlights that peptides targeting the intracellular trafficking of melanocytes are candidate molecules to tune pigmentation in health and disease.

## 1. Introduction

The skin plays a fundamental protective function against solar ultraviolet damage, preventing skin cancers such as melanoma. The outer layer of the skin or epidermis comprises mainly two cell types, keratinocytes and melanocytes. The melanocytes synthesize melanin, the major pigments in mammals.

Melanin biosynthesis occurs within lysosome-related organelles, called melanosomes. Melanosomes of epidermal melanocytes are classified into four morphologically distinct stages [1], of which biogenesis occurs through different steps. Early stages, or pre-melanosomes, originate from the endocytic pathway where vacuolar early sorting endosomes (stage I) form physiological amyloid fibrils organized in parallel arrays in the lumen of stage II [2,3]. Melanins start to be synthesized in stage II and are deposited onto the fibrils, resulting in their thickening and blackening (stage III) until the melanosome lumen is filled (stage IV) [3]. Ultimately, the pigmented melanosomes are transferred to keratinocytes [4], redistributing melanin throughout the epidermis.

Skin melanocytes adapt their intracellular trafficking pathways to ensure melanin biosynthesis within melanosomes. Melanocytic (or melanosome)-specific cargo, such as melanogenic enzymes (tyrosinase (TYR) and related proteins (e.g., TYRP1)), transporters (OCA2) or co-factors (ATP7A), reaches stage I/vacuolar early sorting endosomes before being sorted into tubular recycling endosomal transport intermediates that fuse with melanosomes [5]. Among the proteins controlling the endosome-to-melanosome trafficking pathways, the heterotetrameric adaptor protein (AP)-1 and the microtubule motor KIF13A play a critical function [6]. AP-1 recognizes dileucine motifs in TYR and TYRP1 cytosolic tails allowing, likely, their sorting into nascent recycling endosomal tubules [6,7] that emerge from the stage I pre-melanosome/vacuolar early sorting endosomes and are then transported by KIF13A towards the melanosomes [8,9]. The β1-adaptin subunit of AP-1 interacts directly with KIF13A [6,10] and this couple orchestrates the endosomal sorting, transport, and delivery of melanogenic cargo (e.g., TYRP1) required for melanosome maturation and melanocyte pigmentation [6].

Skin whitening products alleviate pigmentation for the clinical treatment of pigmentary disorders (melasma, senile lentigo, post-inflammatory hyperpigmentation) and cosmetic purposes (even or lighter complexion). Most of the whitening agents tend to modulate melanin production by targeting the TYR pathway [11,12]. For instance, hydroquinone and its derivatives (e.g., hydrocortisone, arbutin, kojic acid) function as TYR inhibitors and decrease the melanin production by 20 to 50% in human normal melanocytes or melanoma cell lines in culture or skin equivalents. Of note, those molecules in combination do not show synergistic effects [13] and are often associated with cytotoxicity and side effects [12].

An alternative in reducing skin pigmentation is to impair the biogenesis and/or maturation of melanosomes; these are processes that also contribute to the multidrug resistance mechanisms operating in melanoma [14,15]. Here we report the design and effects of short AP-1-derived peptides that impair the interaction of KIF13A with the β1-adaptin subunit. These peptides impair melanosome maturation and consistently reduce intracellular melanin production by MNT-1 cells (highly pigmented human non-metastatic melanoma cells) and by human pigmented synthetic epidermis. Such small molecules are certainly valuable candidates for modulating pigmentation.

## 2. Results

### 2.1. β1-Adaptin-Derived Peptides Prevent the AP-1/KIF13A Interaction

**Figure 1:**
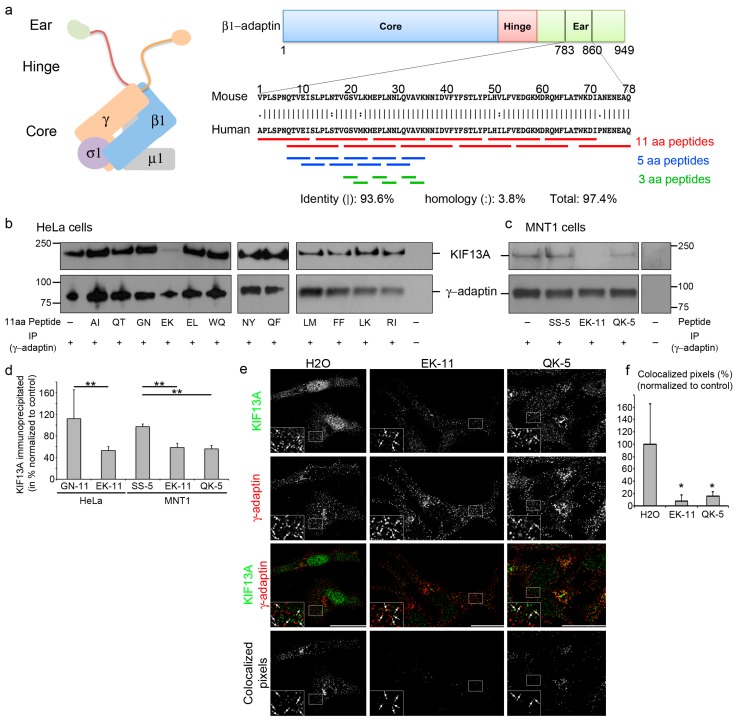
Adaptor protein (AP)-1-derived peptides disrupt the AP1–KIF13A interaction. (**a**) Diagram showing AP-1 complex and β1-adaptin with core (blue), hinge (red), and ear (green) domains, KIF13A binding mouse and human sequences, and corresponding peptides (11aa (red), 5aa (blue), 3aa (green)); (**b,c**) Western blot of γ-adaptin immunoprecipitations of HeLa (**b**) and MNT-1 (**c**) cell lysates incubated or not with 1 µg of peptide using KIF13A (top) or γ-adaptin (bottom) antibodies; (**d**) Quantification of KIF13A revealed γ-adaptin immunoprecipitates; (**e**) Immunofluorescence on MNT-1 cells treated with EK-11, QK-5 or H_2_O as control using KIF13A (1st lane, green) and γ-adaptin antibodies (2nd lane, red). Individual and merged (3rd lane) channels are shown. Co-localization masks (4th lane) showing overlapped pixels (arrows in magnified insets of the boxed area); (**f**) Quantification of co-localized γ-adaptin/KIF13A pixels (excluding nuclei and Golgi area). Data are the average normalized to control and presented as a mean ± standard deviation. * *p* < 0.05; ** *p* < 0.01. Bars, 10 µm.

The AP-1 complex—composed of four subunits (β1- and γ-adaptin, μ1 and σ1 subunits) [16]—binds directly to KIF13A through a 77-amino acid (aa) motif originally defined within the mouse β1-adaptin ear domain (position 783–860) [10]. Interestingly, this domain shows high homology with the human β1-adaptin sequence (Figure 1a). To define molecules blocking the β1-adaptin/KIF13A interaction, 27 individual and partialy overlapping peptides of different lengths (11, 5, or 3aa) were designed from the human β1-adaptin sequence (Figure 1a, Table 1, and Materials and Methods). Given that AP-1 subunits and KIF13A are ubiquitously expressed proteins, we first decided to screen the candidate blocking peptides for their ability to disrupt the AP-1/KIF13A interaction in a non-pigment human cell line such as HeLa cells (Figure 1b). Cell lysates were incubated with the corresponding peptides and subjected to immunoprecipitation using anti-human γ-adaptin antibodies. Among the 11aa peptides (Figure 1a), EK-11 (Table 1) promoted a statistically significant (2-fold) decrease of co-precipitated KIF13A (Figure 1b,d) relative to the adjacent and overlapping GN-11 peptide (used here as a control due to its identical length and similar physicochemical properties; Table 1). We then confirmed in a relevant pigment cell model that incubating MNT-1 cell lysates with EK-11 dramatically reduced the overall amount of precipitated KIF13A (Figure 1c–d). This suggests that EK-11 might represent a potent inhibitor of the AP-1/KIF13A interaction in virtually all human cell types. Interestingly, the last 5 amino acids of the GN-11 peptide correspond to the first 5 amino acids of EK-11, indicating that the second half of EK-11 within the β1-adaptin subunit of AP-1 (named QK-5; Table 1) might overlap the KIF13A binding site and, thus, represent a blocking peptide. Importantly and similarly to EK-11, the incubation of MNT-1 cell lysates with QK-5 decreased the level of precipitated KIF13A (Figure 1c,d) relative to a 5aa SS-5 peptide—found as a non-blocking peptide with comparable physicochemical properties and, therefore, used as a control peptide (Table 1). Therefore, these results indicate that both EK-11 and QK-5 most likely target the same KIF13A binding site; with the QVAVK sequence of the human β1-adaptin (Table 1) corresponding to a putative interaction motif.

### 2.2. β1-Adaptin-Derived Peptides Displace the AP-1/KIF13A Co-Distribution

AP-1 and KIF13A co-distribute at the *trans*-Golgi network or at endosomes dependently of the cell type [6,8,9,10]. Given that EK-11 or QK-5 prevents the AP-1/KIF13A interaction in cell lysates, we reasoned that these peptides could affect AP-1/KIF13A co-localization within cells. Thus, we examined by immunofluorescence microscopy the co-distribution of AP-1 and KIF13A in peptide-treated MNT-1 cells. Control cells incubated with H_2_O—the solvent used to solubilize the lyophilized peptides—displayed a partial co-distribution between the γ-adaptin subunit of AP-1 and KIF13A (Figure 1e, arrows), as previously described [6]. In contrast, EK-11- or QK-5-treated MNT-1 cells (3 days, 10 µM; see Materials and Methods) showed a dramatic decrease in their co-localization relative to control (Figure 1e–f, arrows), suggesting first that those peptides can either diffuse or be actively transported into live cells and, second, that they can displace AP-1 and KIF13A intracellular localization likely by preventing their interaction (Figure 1b–d). Altogether, data show that peptides as short as 5aa efficiently inhibit AP-1/KIF13A interaction and co-localization in vitro and in live cells.

### 2.3. β1-Adaptin-Derived Peptides Decrease Pigmentation And Melanosome Biogenesis

**Figure 2:**
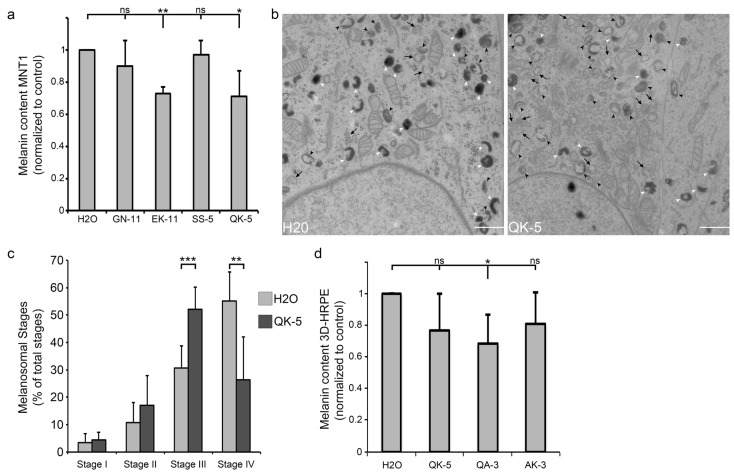
AP-1-derived peptides impact melanin production and melanosome biogenesis. (**a**) Melanin content estimation of MNT-1 cells treated with EK-11, QK-5, GN-11, SS-5 (10 µM), or H_2_O as control; (**b**) Conventional electron microscopy of H_2_O- or QK-5-treated MNT-1 cells. Black arrows: immature melanosomes (stages I/II); black arrowheads: maturing stage III melanosomes; white arrowheads: pigmented stage IV melanosomes. (**c**) Quantification of immature melanosomes (stages I or II) and mature melanosomes (stages III or IV) per condition; *(***d**) Melanin content estimation of 3D-HRPE (phototype VI) treated with 30 µM of QK-5, QA-3, or AK-3 peptides or H_2_O as control. Data are the average presented as a mean ± standard deviation. ns, non-significant; * *p* < 0.05; ** *p* < 0.01; *** *p* < 0.001. Bars, 1 µm.

Given that AP-1 and KIF13A function together along the same endosome-to-melanosome pathway required for pigmentation [6], we further investigated whether incubating pigment cells with those peptides could have an impact on the production of intracellular pigment in MNT-1 cells. Using spectroscopy, EK-11- or QK-5-treated MNT-1 monolayers (3 days, 10 µM; see Materials and Methods) showed a ~30% reduction of their melanin content relative to controls or non-blocking peptides (GN-11, SS-5) (Figure 2a). Among the concentrations of peptides tested (1 µM, 5 µM [17] and 10 µM), only the highest (10 µM; Figure 2a) significantly reduced the melanin content of MNT-1 cells compared to H_2_O-treated cells. Also, incubating MNT-1 cells for 3 days with 10 µM of peptides did not affect their viability relative to controls (H_2_O, 93.5 ± 3.7%; SS-5, 91.1 ± 3.3%; QK-5, 94.8 ± 3.3%), indicating no cytotoxic effect at steady state when melanocytes were not challenged (such as UV exposure). As AP-1 or KIF13A has been shown to equally contribute to the biogenesis and maturation of pigmented melanosomes [6,8], we used conventional electron microscopy to investigate the presence of the different melanosomal stages, their morphology, and their maturation. Control (H_2_O-treated) MNT-1 cells presented few early (stages I/II; black arrows) but numerous late (stage III; black arrowheads) or heavily pigmented (stage IV; white arrowheads) melanosomes (Figure 2b,c and Table 2). In contrast, QK-5-treated MNT-1 cells harbored fewer stage IV while the number of immature stage III increased without significantly impacting the early stages I/II (Figure 2b,c), indicating that peptide treatment consistently slowed stage III to IV melanosome maturation. Together, our results indicate that blocking the AP-1/KIF13A interaction has an impact on the biosynthesis of melanin by delaying the maturation of melanosomes in pigment cells.

### 2.4. β1-Adaptin-Derived Peptides Might Confer Skin Hypopigmentation

To further investigate the impact on pigmentation in a more physiological context, we incubated peptides with 3D human reconstructed pigmented epidermis (3D-HRPE composed of human primary melanocytes of phototype VI and normal human keratinocytes). Hypothesizing that short peptides (5aa or 3aa) might diffuse more efficiently into tissue, 3D-HRPE were incubated (10 days, 30 µM) with QK-5, QA-3 or AK-3—corresponding to the first or last 3 amino acids of QK-5, respectively (Figure 1a and Table 1)—before melanin content estimation. Only QA-3-treated HRPE showed a significant ~30% decrease in the melanin content relative to control (H_2_O) (Figure 2d). Compared to cell monolayers incubated with QK-5 (Figure 2a), QK-5-treated HRPE did not show a robust pigmentation decrease. We reason that the 5aa length of QK-5 was possibly a key criterion affecting its optimal diffusion and action throughout the 3D-tissue. Yet, we did not investigate further whether optimizing the QK-5 peptide might have a significant impact on the melanin production in 3D-HRPE. However, the result indicates that those peptides, and especially QA-3, might be potentially used as skin whitening molecules.

## 3. Discussion

We identified peptides blocking the AP-1/KIF13A interaction and decreasing pigmentation by likely slowing the maturation of melanosomes in MNT-1 cells and synthetic epidermis. The identification and characterization of these peptides certainly open a therapeutic avenue to modulate pigmentation. Drug resistance of melanoma cells can be linked to their ability to form fully pigmented melanosomes [14,15]. It might, therefore, be important to investigate whether such peptides, alone or in combination with other known molecules, may increase melanoma drug sensitivity by decreasing melanosome maturation and melanocyte pigmentation. By knowing the roles of AP-1 and/or KIF13A in several biological processes [8,9,10,16,18,19]—including pigmentation [6]—the peptides might be used to study the intracellular endosomal sorting, transport, and delivery of cargo not only in melanocytes but also in non-pigment cells. Finally, after evaluating peptides for their stability within formulations and skin penetration, these peptides might be used by the cosmetic industry as promising skin whitening agents. However, given the complex cellular environment of human skin, whether these peptides perturb essential intracellular functions occurring in other dermal cells, such as keratinocytes, Langerhans cells, or fibroblasts, must be examined.

In contrast to TYR inhibitors (e.g., hydroquinone and its derivatives) that represent a class of strong whitening agents associated with cytotoxic effects [12], the peptides described here might represent safer whitening molecules. Indeed, delaying melanosome maturation and pigmentation might represent a reversible “whitening” mechanism that would return back to normal in the absence of the peptides. Of interest, treatment of MNT-1 cells with AP-1- or KIF13A-siRNAs blocks melanosome maturation by accumulating immature unpigmented pre-melanosomes that do not progress to the pigmented stages [6,8]. Therefore, the probable delay in melanosome maturation—instead of a complete block (siRNAs)—observed here in peptide-treated cells suggests that AP-1 and/or KIF13A play additional and unique pigmentary-related functions that remain unknown. Thus, these peptides might allow delineating such subtle cellular functions.

In this study, we did not investigate whether the peptides enter the cells by diffusion or active process. Very few peptides, called cell-penetrating peptides, have the intrinsic capacity to cross the plasma membrane of living cells [20] and their mode of entry remains poorly understood. Whether the peptides described here belong to such a family is not known. Yet, and in addition to their capacity to prevent protein interaction in vitro, the decreased co-localization between AP-1 and KIF13A in the cells suggests that the peptides are efficiently internalized. Incubating the cell monolayer with 10 µM of peptides was sufficient to significantly reduce pigmentation; however, and given that likely not all peptides are internalized, the intracellular concentration of peptides within the cells, even if not measured here, should be lower. Interestingly, the decrease in pigmentation measured upon incubation with peptides is similar to the one observed in AP-1- or KIF13A-siRNA depleted MNT-1 cells [6,8], showing that 10 µM corresponds to the lowest concentration associated with the maximum expected effect in cells.

Among the candidate blocking peptides, QA-3 decreased more efficiently the pigmentation in reconstructed epidermis than QK-5, suggesting that QA-3 might thus represent the minimal KIF13A binding site within the β1-adaptin subunit of AP-1. Of note, the shorter QA-3 peptide (molecular weight: 352.4 g/mol) might diffuse more efficiently into the synthetic tissue than its longer relative QK-5 (molecular weight: 543.7 g/mol). In addition, QK-5 contained 40% hydrophobic residues (two valine) whereas QA-3 included only one valine residue. Therefore, the hydrophilic properties of QA-3 might reflect an increased efficiency for entering and diffusing into cells within the synthetic tissue. Further studies might investigate whether optimizing and/or conjugating those peptides could allow lowering their concentration for transdermal delivery while preserving their efficiency of action.

## 4. Materials and Methods

### 4.1. Peptide Design, Synthesis, and Preparation

Previous work [10] defined the binding domain for mouse KIF13A on β1-adaptin subunit of the mouse AP-1 protein complex as a 77aa region located between the 783rd and 860S aa positions. The homologous sequence of the human β1-adaptin subunit was identified (DNA Strider software) and corresponded to a 77aa region (786–863) sharing >97.4% homology with the mouse domain. The 77aa human sequence (786–860) was used to design 12 peptides of 11aa sharing 5aa with the adjacent peptides (Table 1 and Figure 1a, red bars). Then, 9 overlapping pentapeptides covering and surrounding the 11aa peptide candidates were designed (Table 1 and Figure 1a, blue bars). The selected pentapeptide candidates were divided into two tripeptides with one overlapping aa (Table 1 and Figure 1a, green bars). Lyophilized peptides were synthesized by Proteogenix (Oberhausbergen, France) and diluted either in H_2_O or in dimethyl sulfoxide (DMSO)/H_2_O (1 v/3 v) according to manufacturer’s recommendations.

### 4.2. Cell Culture

HeLa cells were maintained in DMEM supplemented with 10% FBS and antibiotics (Thermo Fisher Scientific, Waltham, MA, USA) as described [9]. MNT-1 cells, a highly pigmented melanoma cell line resembling normal melanocyte pigment expression, were maintained in DMEM supplemented with 10% AIM-V medium, 20% FBS, non-essential amino acids, sodium pyruvate, and antibiotics (Thermo Fisher Scientific) as described [6,8].

### 4.3. Protein Extraction, Immunoprecipitation, and Western Blot Analysis

Cells at 80% of confluence were washed in cold PBS and lysed on ice in lysis buffer (50 mM Tris, 150 mM NaCl, 0.1% Triton X-100, 10 mM EDTA, pH 7.2, and protease inhibitor cocktail (Merck, Darmstatd, Germany)). Lysates were incubated overnight with 1 µg of peptides at 4 °C under rotation. Lysates were first pre-cleared using protein G agarose beads (Invitrogen, Carlsbad, CA, USA) for 1 h at 4 °C under rotation. Supernatants were collected and incubated with protein G agarose beads pre-loaded with 1 µg mouse monoclonal IgG2b anti-CD9 (Abcam, Cambridge, UK, ab19761) for 1 h at 4 °C under rotation. Pre-cleared supernatants were incubated with beads pre-coated with 1 µg mouse monoclonal IgG2b anti–γ-adaptin (Sigma-Aldrich, St. Louis, MO, USA, clone 100/3) for 2 h at 4°C under rotation. Mouse monoclonal IgG2b anti-CD9 (1 µg) was used as the negative control for immunoprecipitation. Beads were washed 5 times in cold lysis buffer and 3 times in cold PBS; the immunoprecipitated proteins bound to the beads were incubated in sample buffer with reducing agent (Invitrogen), boiled for 10 min at 95 °C, and fractionated by SDS-PAGE using Nupage (3–8%) Tris-acetate gels (Invitrogen). Transfer was realized on PVDF membrane (Millipore, Burlington, MA, USA) probed with mouse monoclonal anti–γ-adaptin and rabbit polyclonal to KIF13A (Bethyl Laboratory, Inc., Montgomery, TX, USA, A301-077A). At least three independent experiments were performed. Densitometry analyses were carried out using ImageJ software (NIH) with background subtraction to have accurate fold comparisons within and between experiments. Results presented in Figure 1d: HeLa: GN-11, 112 ± 53%; EK-11, 53 ± 7%; MNT1: GN-11, 97 ± 5%; EK-11, 58 ± 8%; QK-5, 56 ± 6%.

### 4.4. Immunofluorescence

MNT-1 cells grown on coverslips were incubated for 3 days in medium containing peptides (10 µM). Media containing peptides were renewed every day. Coverslips fixed in 100% glacial methanol for 5 s were washed 5 times in distilled water and incubated in PBS/1 mg/mL BSA (incubation buffer, IB). Fixed cells were incubated for 1 h with mouse monoclonal anti–γ-adaptin (Sigma-Aldrich, clone 100/3) and rabbit polyclonal to KIF13A (Bethyl Laboratory, Inc., A301-077A) diluted in IB, washed three times in IB, and incubated with the corresponding secondary antibody (Alexa Fluor, Invitrogen) for 30 min. Cells were washed twice in IB and once in PBS before mounting the coverslips in DABCO medium (Invitrogen) and examining on an Eclipse 80i Upright Microscope (Nikon, Tokyo, Japan) equipped with a CoolSNAP HQ2 CCD Camera, a Piezo Flexure Objective Scanner and 100× Plan Apo objective (1.4 NA CFI). 

### 4.5. Co-Localization Analysis

Co-localization analysis was performed using ImageJ software. Co-localization masks were generated using the Co-localization Plugin. Pixels for which the intensity of the overlay exceeded the intensity of the individual channels were considered as co-localizing pixels. Identical intracellular areas were defined and analyzed for quantification excluding the nuclei and Golgi area. Two independent experiments were performed on more than 10 cells. Results presented in Figure 1f: H_2_O, 100 ± 66%; EK-11, 8 ± 10%; QK-5, 16 ± 7%.

### 4.6. Conventional Electron Microscopy

Control- and peptide-treated MNT-1 cells grown on coverslips for 3 days—in media containing or not 10 µM peptides (media were renewed every day)—were fixed with 2.5% glutaraldehyde/0.1 M cacodylate buffer, pH 7.2, post-fixed with 1% OsO_4_ supplemented with 1.5% potassium ferrocyanure, dehydrated in ethanol, and embedded in Epon. Ultrathin sectioning was performed with an ultramicrotome (UCT, Leica, Wetzlar, Germany). Sections were counter-stained with uranyl acetate and lead citrate and electron micrographs were acquired under electron microscopes (Philips CM120 or Tecnai Spirit G2; FEI, Eindhoven, The Netherlands) equipped with a numeric camera (Keen View; Soft Imaging System) or 4k CCD camera (Quemesa, Olympus, Muenster, Germany). Melanosome stages from I to IV were quantified based on their morphology and melanin content (see Table 2 and [3]). Two independent quantifications by two different authors were performed on more than 100 structures. Results presented in Figure 2c: Stage I: H_2_O, 4 ± 3%; QK-5, 5 ± 3%; Stage II: H_2_O, 11 ± 7%; QK-5, 17 ± 11%; Stage III: H_2_O, 31 ± 8%; QK-5, 52 ± 8%; Stage IV: H_2_O, 55 ± 11%; QK-5, 26 ± 16%.

### 4.7. Melanin Assay

MNT-1 cells (1 × 10^5^) at day 1 were seeded in 6-well plates. Cells were grown in medium containing 1, 5, or 10 µM peptides (day 2 to 4). Medium was renewed every day. Cells were mechanically broken by sonication (day 5) in 50 mM Tris-HCl, 2 mM EDTA, 150 mM NaCl, 1 mM dithiothreitol, and protease inhibitors, pH 7.4. Cell lysates were centrifuged at 13,000 rpm for 15 min at 4 °C. The pellet was rinsed once in ethanol/ether (1:1) and dissolved in 2 M NaOH/20% dimethyl sulfoxide at 60 °C to collect melanin pigment that was measured by optical density (492 nm). Three to seven independent experiments were performed. Results in Figure 2a: GN-11, 0.9 ± 0.16; EK-11, 0.73 ± 0.04; SS-5, 0.97 ± 0.09; QK-5, 0.71 ± 0.16. Reconstructed human pigmented epidermis of phototype VI (3D-HRPE, Sterlab, France) were received at day 10 of culture and incubated (day 11 to 21) with manufacturer’s growth medium supplemented with peptides (30 µM). Medium was renewed every day. The epidermis (day 21) was separated from the insert and digested in Solvable (400 µL, 98 °C, 1 h; PerkinElmer) as described [21]. The melanin content was measured by optical density at 492 nm. Three independent experiments were performed by two independent authors. Results in Figure 2d: QK-5, 0.76 ± 0.23; QA-3, 0.69 ± 0.18; AK-3, 0.80 ± 0.20.

### 4.8. Statistical Analysis

Statistical differences were evaluated between 2 means by Student’s *t*-test adapted for small number of samples, by unpaired Student’s *t*-test on GraphPad Prism when samples were independent, or between >2 means with one-way non-parametric ANOVA test followed by a Bonferroni corrected *t*-test. A *p*-value *p* < 0.05 was considered as statistically significant.

## Figures and Tables

**Table 1:** Peptides derived from the human β1-adaptin sequence (position 783–860) and their respective solvent used in this study.

Peptide (Solvent)	11aa Peptide Sequences	Peptide (Solvent)	5aa Peptide Sequences	Peptide (Solvent)	3aa Peptide Sequences
AI-11 (H_2_O)	APLSPNQTVEI	QI-5 (H_2_O)	QTVEI	GV-3 (H_2_O)	GSV
QT-11 (H_2_O)	QTVEISLPLST	EP-5 (H_2_O)	EISLP	VK-3 (H_2_O)	VMK
LK-11 (DMSO)	LPLSTVGSVMK	LT-5 (H_2_O)	LPLST	EL-3 (H_2_O)	EPL
GN-11 (H_2_O)	GSVMKMEPLNN	SS-5 (H_2_O)	STVGS	LN-3 (H_2_O)	LNN
EK-11 (H_2_O)	EPLNNLQVAVK	GK-5 (H_2_O)	GSVMK	QA-3 (H_2_O)	QVA
QF-11 (DMSO)	QVAVKNNIDVF	MP-5 (H_2_O)	MKMEP	AK-3 (H_2_O)	AVK
NY-11 (DMSO)	NIDVFYFSTLY	EN-5 (H_2_O)	EPLNN		
FF-11 (DMSO)	FSTLYPLHILF	NV-5 (H_2_O)	NNLQV		
LM-11 (DMSO)	LHILFVEDGKM	QK-5 (H_2_O)	QVAVK		
EL-11 (H_2_O)	EDGKMDRQMFL				
RI-11 (DMSO)	RQMFLATWKDI				
WQ-11 (H_2_O)	WKDIPNENEAQ				

**Table 2:** Ultrastructural characteristics of melanosome stages.

Stage	Criteria
I	Spherical, no melanin, presence of a planar clathrin coat, and few intraluminal vesicles
II	Oval, no melanin, presence of internal fibrils
III	Oval, moderate deposits of melanin onto internal fibrils
IV	Oval, intense deposits of melanin onto internal fibrils

## Abbreviations

TYRP1	tyrosinase-related protein 1
AP-1	adaptor protein-1
aa	amino acid

## References

[1] Seiji M., Fitzpatrick T.M., Simpson R.T., Birbeck M.S.C. (1963). Chemical composition and terminology of specialized organelles (melanosomes and melanin granules) in mammalian melanocytes. Nature.

[2] Hurbain I., Geerts W.J., Boudier T., Marco S., Verkleij A.J., Marks M.S., Raposo G. (2008). Electron tomography of early melanosomes: Implications for melanogenesis and the generation of fibrillar amyloid sheets. Proc. Natl. Acad. Sci. USA.

[3] Raposo G., Tenza D., Murphy D.M., Berson J.F., Marks M.S. (2001). Distinct protein sorting and localization to premelanosomes, melanosomes, and lysosomes in pigmented melanocytic cells. J. Cell Biol..

[4] Wu X., Hammer J.A. (2014). Melanosome transfer: It is best to give and receive. Curr. Opin. Cell Biol..

[5] Sitaram A., Marks M.S. (2012). Mechanisms of protein delivery to melanosomes in pigment cells. Physiology.

[6] Delevoye C., Hurbain I., Tenza D., Sibarita J.B., Uzan-Gafsou S., Ohno H., Geerts W.J., Verkleij A.J., Salamero J., Marks M.S. (2009). AP-1 and KIF13A coordinate endosomal sorting and positioning during melanosome biogenesis. J. Cell Biol..

[7] Theos A.C., Tenza D., Martina J.A., Hurbain I., Peden A.A., Sviderskaya E.V., Stewart A., Robinson M.S., Bennett D.C., Cutler D.F. (2005). Functions of adaptor protein (AP)-3 and AP-1 in tyrosinase sorting from endosomes to melanosomes. Mol. Biol. Cell.

[8] Delevoye C., Heiligenstein X., Ripoll L., Gilles-Marsens F., Dennis M.K., Linares R.A., Derman L., Gokhale A., Morel E., Faundez V. (2016). BLOC-1 brings together the actin and microtubule cytoskeletons to generate recycling endosomes. Curr. Biol..

[9] Delevoye C., Miserey-Lenkei S., Montagnac G., Gilles-Marsens F., Paul-Gilloteaux P., Giordano F., Waharte F., Marks M.S., Goud B., Raposo G. (2014). Recycling endosome tubule morphogenesis from sorting endosomes requires the kinesin motor KIF13A. Cell Rep..

[10] Nakagawa T., Setou M., Seog D., Ogasawara K., Dohmae N., Takio K., Hirokawa N. (2000). A novel motor, KIF13A, transports mannose-6-phosphate receptor to plasma membrane through direct interaction with AP-1 complex. Cell.

[11] Ebanks J.P., Wickett R.R., Boissy R.E. (2009). Mechanisms regulating skin pigmentation: The rise and fall of complexion coloration. Int. J. Mol. Sci..

[12] Solano F., Briganti S., Picardo M., Ghanem G. (2006). Hypopigmenting agents: An updated review on biological, chemical and clinical aspects. Pigment Cell Res..

[13] Yoshimura K., Tsukamoto K., Okazaki M., Virador V.M., Lei T.C., Suzuki Y., Uchida G., Kitano Y., Harii K. (2001). Effects of all-trans retinoic acid on melanogenesis in pigmented skin equivalents and monolayer culture of melanocytes. J. Dermatol. Sci..

[14] Huang Z.M., Chinen M., Chang P.J., Xie T., Zhong L., Demetriou S., Patel M.P., Scherzer R., Sviderskaya E.V., Bennett D.C. (2012). Targeting protein-trafficking pathways alters melanoma treatment sensitivity. Proc. Natl. Acad. Sci. USA.

[15] Xie T., Nguyen T., Hupe M., Wei M.L. (2009). Multidrug resistance decreases with mutations of melanosomal regulatory genes. Cancer Res..

[16] Nakatsu F., Hase K., Ohno H. (2014). The role of the clathrin adaptor AP-1: Polarized sorting and beyond. Membranes.

[17] Campagne C., Ripoll L., Gilles-Marsens F., Raposo G., Delevoye C. (2018). Melanin estimation of peptide-treated MNT-1 cells (1 µM and 5 µM peptide concentration).

[18] Zhou R., Niwa S., Guillaud L., Tong Y., Hirokawa N. (2013). A molecular motor, KIF13A, controls anxiety by transporting the serotonin type 1A receptor. Cell Rep..

[19] Ramos-Nascimento A., Kellen B., Ferreira F., Alenquer M., Vale-Costa S., Raposo G., Delevoye C., Amorim M.J. (2017). KIF13A mediates trafficking of influenza A virus ribonucleoproteins. J. Cell Sci..

[20] Bechara C., Sagan S. (2013). Cell-penetrating peptides: 20 years later, where do we stand?. FEBS Lett..

[21] Lo Cicero A., Delevoye C., Gilles-Marsens F., Loew D., Dingli F., Guere C., Andre N., Vie K., van Niel G., Raposo G. (2015). Exosomes released by keratinocytes modulate melanocyte pigmentation. Nat. Commun..

